# Dynamic Changes of the Gut Microbiota in Preterm Infants With Different Gestational Age

**DOI:** 10.3389/fmicb.2022.923273

**Published:** 2022-06-30

**Authors:** Qiong Jia, Xue Yu, Yanmei Chang, Yanxia You, Zekun Chen, Ying Wang, Bin Liu, Lijun Chen, Defu Ma, Yan Xing, Xiaomei Tong

**Affiliations:** ^1^Department of Pediatrics, Peking University Third Hospital, Beijing, China; ^2^School of Public Health, Peking University Health Science Center, Beijing, China; ^3^National Engineering Center of Dairy for Maternal and Child Health, Beijing Sanyuan Foods Co., Ltd., Beijing, China

**Keywords:** gut microbiota, gestational age, *Klebsiella*, *Enterococcus*, *Bifidobacterium*

## Abstract

The gut microbiota plays a key role in the pathogenesis of diseases affecting preterm infants and gestational age is one of the important factors which affect the gut microbiota of infants. To determine the characteristics of the gut microbiota in preterm infants of different gestational ages from birth to 1 year after birth, we collected 622 fecal samples from neonates of different gestational ages at different time points after birth. According to the gestational ages, the samples were divided into four groups, extremely preterm, very preterm, moderate to late preterm, and term group. Meconium and fecal samples at day 14, 28, 120, and 365 after birth were collected. 16S rRNA sequencing was performed and the composition and structure of the gut microbiota in preterm infants of different gestational age was compared with that of term infants. In our study, alpha diversity of meconium in extremely preterm group was higher than very preterm group, moderate to late preterm group and term group and alpha diversity of meconium in preterm group was decreased with increasing of gestational age. At day 14 to day 120 after birth, alpha diversity of term and moderate to late preterm group were significantly higher than other two preterm groups. However, moderate to late preterm group owned the highest alpha diversity which was higher than term group at day 365 after birth. Besides, the results shown the duration of opportunistic pathogen such as *Klebsiella* and *Enterococcus* which dominant colonization was different in different gestational age groups. As well as the probiotics, such as *Bifidobacterium*, which abundance enriched at different time point in different gestational age groups. We profiled the features of dynamic changes of gut microbiome from different gestational ages infants. The results of our research provide new insights for individualized interventions of specific microbes of preterm infants with different gestational ages at different time points after birth.

## Introduction

Preterm births are a major public health issue. A study (Deng et al., 2021) used national monitoring data from China’s National Maternal Near Miss Surveillance System (NMNMSS) to estimate the preterm birth rate and trends between 2012 and 2018 in China. The results showed that among the babies of 9 million women with at least one live baby, 6.1% were preterm and the overall preterm birth rate increased from 5.9% in 2012 to 6.4% in 2018. Recently, with the development of medical technology the survival rate of some preterm infants has increased. From 1990 to 2018, the global annual neonatal mortality rate has decreased by 51% (Yu et al., 2021). Some extremely preterm infants [gestational age (GA) < 28 weeks] receive good medical treatment and grow up. However, preterm neonates still face more challenges than term infants because of their immaturity, which may confer an increased risk of morbidity and mortality and lead to diseases, such as necrotizing enterocolitis (NEC) with a mortality rate of 10–30% (Hui et al., 2021), sepsis (El Manouni El Hassani et al., 2021) etc. (Lee et al., 2020). Lots of studies have indicated that many preterm infant diseases are highly associated with the gut microbiota. Stewart et al. (2013) reported that babies who developed NEC owned lower diversity of gut microbiota. Olm et al. (2019) performed metagenomic analysis of fecal samples from premature infants and found replication of all bacteria were significantly higher 2 days before NEC diagnosis, meanwhile samples collected before NEC diagnosis harbored significantly more *Klebsiella*. For late-onset neonatal sepsis (LONS), pathogens isolated in cases of LONS are usually existed in the gut microbiota (Stewart et al., 2017). Beghetti et al. (2022) suggested that early *Bifidobacterium* deficiency seems to be a negative biomarker of adverse neurological outcomes in very low birth weight infants through a pilot observational study.

Gestational age is one of factors which influence the gut microbiota in infants (Aguilar-Lopez et al., 2021). Compared with term infants, preterm infants have altered gut microbiota. Studies have shown that the meconium of preterm infants has lower diversity, reduced number of *Bifidobacteria*, and increased number of *Enterobacter, Enterococcus, and Staphylococcus*, compared with that of term infants (Korpela et al., 2018). Although numerous studies indicate that intestinal bacteria play an essential role in the pathogenesis of NEC, early onset neonatal sepsis (EONS), and LONS, no single bacterial species has been consistently identified as the causative agent. Several microorganisms have been implicated in NEC, primarily from the phylum *Firmicutes* and *Proteobacteria*. Specifically, an increased abundance of *Clostridium spp.* and associated toxins have been observed in the stools of neonates with NEC (Lee et al., 2020). However, few studies have investigated the dynamic changes in the gut microbiota of preterm infants for over half a year.

In this study, we collected meconium and fecal samples from preterm infants with different GA and term infants from the neonatal period until 1 year after birth, and analyzed the microbiota composition using 16S rRNA sequencing. Subsequently, we compared the gut microbiota composition of different GA preterm infants with that of term infants at different time points after birth. Finally, we profiled the dynamic changes in the gut microbiota of preterm infants between different GA and different ages after birth.

## Materials and Methods

### Study Cohort

Informed consent was obtained from the parents of the enrolled newborns. The study was conducted in accordance with the Declaration of Helsinki, and the protocol was approved by the Ethics Committee of Peking University Third Hospital (No. M2017183).

Term infants were recruited at the Department of Obstetrics of Peking University Third Hospital as controls. Preterm infants (GA: 24–37 weeks) were recruited at the neonatal intensive care unit (NICU) of the Department of Pediatrics of Peking University Third Hospital.

**FIGURE 1:**
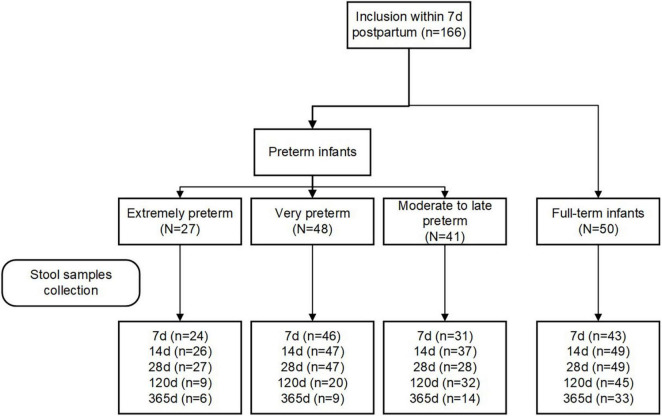
Flow chart of the study cohort.

Eligibility criteria included women aged 18 years and older with the intention to breastfeed for at least fourth months postpartum. Women who delivered infants with congenital malformations, genetic diseases, or those who required surgery were excluded. Participants were grouped into four groups according to their infants’ GA at birth: extremely preterm (< 28 weeks; EP), very preterm (28–32 weeks; VP), moderate to late preterm (32–37 weeks; MP), and full-term (37–42 weeks, T). The study flowchart is depicted in Figure 1.

Baseline information of both the mothers and infants, which included maternal factors (age, weight, height, and mode of delivery) and the infants’ gestational age, birth weight, length, head circumference, Apgar score at 1 min and 5 min after birth, and disease, was collected through the electronic medical record system.

### Sample Collection

Meconium samples were collected the of every neonate during first 2–3 days, for some preterm babies, within 1 week after birth. And other fecal samples were collected at days 14, 28, 120, and 1 year after birth. Using a collection tube containing 8 mL of DNA stabilizer reagent (PSP Spin Stool DNA Plus Kit, STRATEC Biomedical AG, Birkenfeld, Germany). The fecal samples of preterm infants were obtained as part of routine care in the NICU by nurses and stored at −80^°^C. At home, fecal samples were collected by the parents of the infants. Home-collected samples were immediately stored at −20°C and delivered to the hospital on ice bags or dry ice and stored at −80°C.

### 16S rRNA Sequencing

DNA was extracted from each fecal sample using QIAamp Fast DNA Stool Mini Kit (Qiagen, Germany), following the manufacturer’s protocol, which included preceding bead beating. The V3–V4 hypervariable region of the 16S rDNA gene was amplified using the universal primer pair 341F (5′-CCTACGGGAGGCAGCAG-3′) and 806R (5′-GGACTACHVGGGTWTCTAAT-3′). The PCR amplification conditions were as follows: initial denaturation at 98°C for 1 min, followed by 30 cycles at 98°C for 10 s, 50°C for 30 s, and 72°C for 30 s, and a final extension step at 72°C for 5 min. The PCR products (approximately 200 bp) were separated by electrophoresis in agarose gels (2%, w/v), purified with Qiagen Gel Extraction Kit (Qiagen, Germany), and then pooled at equal concentrations. Pyrosequencing was conducted on an Illumina Hiseq 2500 (Illumina, United States).

### Sequence Data Processing and Statistics

All paired-end reads were assigned to each sample based on their unique barcodes and then merged using Fast Length Adjustment of Short reads (FLASH) software. Merged sequences of poor quality were identified and removed by Quantitative Insights into Microbial Ecology (QIIME2) quality filters under specific filtering conditions (Q-score ≥ 25). Chimera removal and dereplication of the reads were performed using USEARCH. Unique sequences in the high-quality merged sequences of each sample were assigned to the same operational taxonomic units (OTUs) at an identity threshold of 97% similarity. A representative sequence was screened for each OTU and used to assign taxonomic composition by the classifier approach in the Ribosomal Database Project database (Wang et al., 2007; Cole et al., 2009).

All statistical analyses were performed using R version 4.0.3. Alpha-diversity (Shannon index) based on the genus level abundance profile was calculated using the Vegan package and compared by using Wilcoxon rank-sum test. Anosim analysis of beta-diversity was calculated based on unweighted unifrac distances using the Vegan package, and principal co-ordinates analysis (PCoA) plot were performed using the ggplot2 package. Linear discriminant analysis (LDA) was applied to determine the most discriminant taxa between the groups, and the size effect cutoff was 2.0. In order to compare the microbiota community structure across four groups, Kruskal–Wallis test was used, and *p*-values were corrected for multiple testing using the Benjamin and Hochberg method.

### Clinical Data Analysis

EpiData 3.1 was used for parallel double entry of demographic and clinical data. Baseline characteristics of the mothers and infants are presented as the mean ± SD for continuous variables and frequencies for categorical variables. A *p*-value of < 0.05 was considered statistically significant.

## Results

### Demographic and Clinical Information

We included 116 preterm (27 EP, 48 VP, and 41 MP) and 50 term infants in this study. The study flowchart is depicted in Figure 1. We analyzed the basic characteristics of infants and their mothers (Table 1) and found that gestational weight gain, conception method (spontaneous), delivery mode (C-section), multiple births (singleton), mother’s high school education or less, hypertension, and pre-eclampsia during pregnancy were significantly different among the four groups (all *P* < 0.05). Because of the different GA at delivery, the birth weight, birth length, head circumference, and Apgar score of the neonates in the four groups at 1 min and 5 min after birth were markedly different (all *P* < 0.001).

**TABLE 1:** Clinical manifestation of mothers and neonates.

	Term	Moderate-late preterm	Very preterm	Extremely preterm	*P*
Maternal age (year)	33.5 ± 4.13	33.4 ± 3.8	33.23 ± 5.21	32.81 ± 3.83	0.926
Maternal pre-pregnancy BMI (kg/m^2^)	22.73 ± 3.38	22.82 ± 2.84	23.06 ± 4.44	23.03 ± 3.46	0.971
Gestational weight gain (kg)	13.65 ± 4.25	13.89 ± 4.87	10 ± 4.48	10.33 ± 5.28	< 0.001
Conception method (*n*, %)					
Spontaneous	38(76)	28(71.8)	28(59.6)	9(40.9)	0.021
Assisted	12(24)	11(28.2)	19(40.4)	13(59.1)	
Delivery mode (*n*, %)					
C-section	24(48)	35(87.5)	36(75)	5(19.2)	< 0.001
Vagina	26(52)	5(12.5)	12(25)	21(80.8)	
Multiple births (*n*, %)					
Singleton	8(96)	14(35)	28(58.3)	11(42.3)	< 0.001
Twins	2(4)	26(65)	20(41.7)	15(57.7)	
Maternal parity (*n*, %)					
1	33(66)	28(70)	31(64.6)	19(73.1)	00.869
> 1	17(34)	12(30)	17(35.4)	7(26.9)	
Mother’s education (*n*, %)					
High school or less	0(0)	3(7.5)	15(31.3)	4(14.8)	< 0.001
Undergraduate	27(54)	27(67.5)	25(52.1)	18(66.7)	
Postgraduate or above	23(46)	10(25)	8(16.7)	5(18.5)	
Diseases during pregnancy (*n*, %)					
Anemia	6(12)	5(12.8)	5(11.6)	2(9.1)	0.978
Diabetes	18(36)	11(28.2)	18(41.9)	5(22.7)	0.375
Hypertension	1(2)	8(20.5)	11(25.6)	2(9.1)	0.006
Pre-eclampsia	0(0)	8(20.5)	9(20.9)	1(4.5)	0.003
Infant sex (*n*, %)					
Boys	34(68)	23(57.5)	23(47.9)	15(57.7)	0.255
Girls	16(32)	17(42.5)	25(52.1)	11(42.3)	
Gestational age at delivery (week)	38.78 ± 1.15	34.03 ± 1.07	30.02 ± 1.41	26.19 ± 0.69	< 0.001
Birth weight (g)	3263.2 ± 480.4	1991.25 ± 425.7	1341.81 ± 339.7	921.92 ± 136.03	< 0.001
Birth length (cm)	48.82 ± 3.8	43.2 ± 3.23	38.13 ± 3.47	34.9 ± 2.47	< 0.001
Head circumference (cm)	34.26 ± 1.64	31.05 ± 1.84	27.65 ± 2.37	24.54 ± 1.79	< 0.001
Apgar score at 1 min	9.9 ± 0.51	9.6 ± 0.93	8.92 ± 1.51	6.65 ± 2.02	< 0.001
Apgar score at 5 min	10 ± 0	9.78 ± 0.73	9.48 ± 0.8	8.38 ± 0.75	< 0.001
NRDS	ND	2(5.3)	17(36.2)	21(87.5)	< 0.001
Pneumonia	ND	4(10.5)	10(21.3)	14(58.3)	< 0.001
PVL	ND	0(0)	6(13.3)	2(9.1)	0.020
PDA	ND	8(21.1)	24(51.1)	16(66.7)	0.001
BPD	ND	0(0)	17(37.8)	22(91.7)	< 0.001
Sepsis	ND	4(10.5)	3(6.5)	5(20.8)	0.220
NEC	ND	0(0)	1(2.1)	0(0)	0.429

Among preterm infants, the number of infants with neonatal respiratory distress syndrome (NRDS), pneumonia, periventricular leukomalacia (PVL), patent ductus arteriosus (PDA), and bronchopulmonary dysplasia (BPD) were remarkably different, and the shorter the GA, the higher the number of newborns with NRDS, pneumonia, and BPD. The number of infants with sepsis and NEC was not significantly different among preterm infants.

For preterm infants, especially preterm infants in NICU, we always use amoxicillin clavulanate potassium, cefoperazone sulbactam, ceftazidime, meropenem, vancomycin for treatment according to the clinical infect manifestations and biochemical tests. Because different infants used different antibiotics for different durations and one infant could use two antibiotics simultaneously, we did not perform the statistics on antibiotics using.

We collected the feeding pattern through medical records and questionnaires, furthermore analyzed the feeding pattern of each group (Table 2).

**TABLE 2:** Feeding pattern.

Time	Feeding patterns	Term		Moderate-late preterm		Very preterm		Extremely preterm	
		Counts	Percentage	Counts	Percentage	Counts	Percentage	Counts	Percentage
Within 45 days after birth	Formula feeding	1	4.55%	4	22.22%	0	0.00%	0	0.00%
	Mixed feeding	9	40.91%	3	16.67%	1	33.33%	0	0.00%
	Brest feeding	12	54.55%	11	61.11%	2	66.67%	0	0.00%
Within 100 days after birth	Formula feeding	1	4.17%	0	0.00%	3	21.43%	0	0.00%
	Mixed feeding	11	45.83%	4	66.67%	7	50.00%	0	0.00%
	Brest feeding	12	50.00%	2	33.33%	4	28.57%	4	100.00%
6 months after birth	Formula feeding	4	10.00%	1	3.57%	5	26.32%	1	14.29%
	Mixed feeding	13	32.50%	18	64.29%	7	36.84%	4	57.14%
	Brest feeding	23	57.50%	9	32.14%	7	36.84%	2	28.57%
1 year after birth	Formula feeding	6	20.69%	0	0.00%	10	55.56%	3	37.50%
	Mixed feeding	15	51.72%	11	73.33%	6	33.33%	3	37.50%
	Brest feeding	8	27.59%	4	26.67%	2	11.11%	2	25.00%

### Structure of the Gut Microbiota in Preterm Infants With Different Gestational Age

We collected fecal samples at each time point from as many infants as possible, and obtained a total of 622 samples. Excluding samples with poor sequencing quality, 617 samples (Table 3) were available for sequencing and finally we got 616 sequencing data. A comparison of the results of the gut microbiota composition in preterm infants with different GA was conducted at different days after birth.

**TABLE 3:** Number of fecal samples from each group.

Group	Meconium	Day 14	Day 28	Day 120	Day 365
EP	24	25	26	9	6
VP	46	46	47	20	9
MP	30	36	28	32	14
T	43	49	49	45	33

*EP, extremely preterm (< 28 weeks); VP, very preterm (28–32 weeks); MP, moderate to late preterm (32–37 weeks); T, full-term (37–42 weeks).*

#### Meconium

**FIGURE 2:**
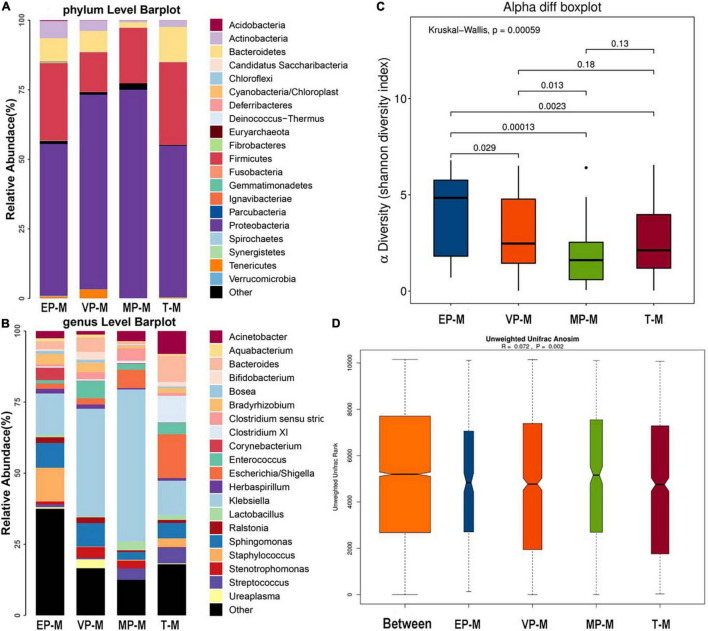
The composition of gut microbiota from neonates with different gestational ages at first day after birth. EP, extremely preterm (<28 weeks); VP, very preterm (28–32 weeks); MP, moderate to late preterm (32–37 weeks); T, full-term (37–42 weeks). **(A)** Comparison of abundance at phylum level. The abscissa is the group name, and the ordinate is the relative abundance of species. Different colors correspond to different species names, and the length of the color block indicates the relative abundance of the species represented by the color block. **(B)** Comparison of abundance at genus level. The abscissa is the group name, and the ordinate is the relative abundance of species. Different colors correspond to different species names, and the length of the color block indicates the relative abundance of the species represented by the color block. **(C)** Shannon diversity index of α diversity. The abscissa represents the groups, and the ordinate represents the Alpha diversity index value. **(D)** Anosim analysis of β diversity. The abscissa represents all samples (Between) and each group, and the ordinate represents the rank of the unifrac distance. When the rank of the Between group is high relative to each of the other groups, it indicates that the between-group difference is greater than the within-group difference. R is between (−1, 1), *R* > 0, indicating that the difference between groups is greater than the difference within the group; *R* < 0, indicating that the difference within the group is greater than the difference between the groups, the reliability of statistical analysis is represented by *P*, *P* < 0.05 indicates statistical significance.

The three most abundant bacteria at the phylum level in each group were Proteobacteria, Firmicutes, and Bacteroidetes (Figure 2A). Although Proteobacteria was both 54% and Firmicutes was nearly 28–30% in meconium of EP and T group, the abundance of the three phyla were significantly different among four groups (Supplementary Table 1). The genera varied greatly among the groups. The top three most abundant genera in the EP group were *Klebsiella* (14.5%), *Staphylococcus* (11.9%), and *Sphingomonas* (8.7%), those in the VP group were *Klebsiella* (37.8%), *Sphingomonas* (8.3%), and *Enterococcus* (6.0%), those in the MP group were *Klebsiella* (53.5%), *Escherichia/Shigella* (6.5%) and *Clostridium* (4.3%), and those in the T group were *Escherichia/Shigella* (15.5%), *Klebsiella* (12.1%), and *Clostridium XI* (9.4%) (Figure 2B). Except *Enterococcus* and *Escherichia/Shigella*, the left genera were significantly different (Supplementary Table 1). Compared with the EP group, the Shannon index of alpha diversity was completely different in the other preterm and term groups (Figure 2C). The Shannon index of EP was higher than other preterm infant groups and term group. The index of analysis of similarities (ANOSIM) that reflected the beta diversity (*R* = 0.072, *P* < 0.05; Figure 2D), indicated that the four groups exhibited significantly different structures of meconium microbiota. *Klebsiella* was nearly about 12–15% in EP and T group lower than VP (38%) group and MP group (53%). *Klebsiella* was the main genus in the meconium of preterm infants. The main genus contributing to the T group was completely different from that of the preterm infant groups. LDA showed that the communities that had a significant impact on the group division were *Bacillus* (order), *Staphylococcaceae* (family), and *Staphylococcus* in the EP group, *Xanthomonadales* (order), *Xanthomonadaceae* (family), and *Stenotrophomonas* in the VP group, *Gammaproteobacteria* (class), *Klebsiella*, and *Enterobacteriaceae* (family) in the MP group, and Bacteroidetes, *Bacteroidia* (class), and *Bacteroidales* (order) in the T group (Supplementary Figure 1).

#### Day 14 After Birth

**FIGURE 3:**
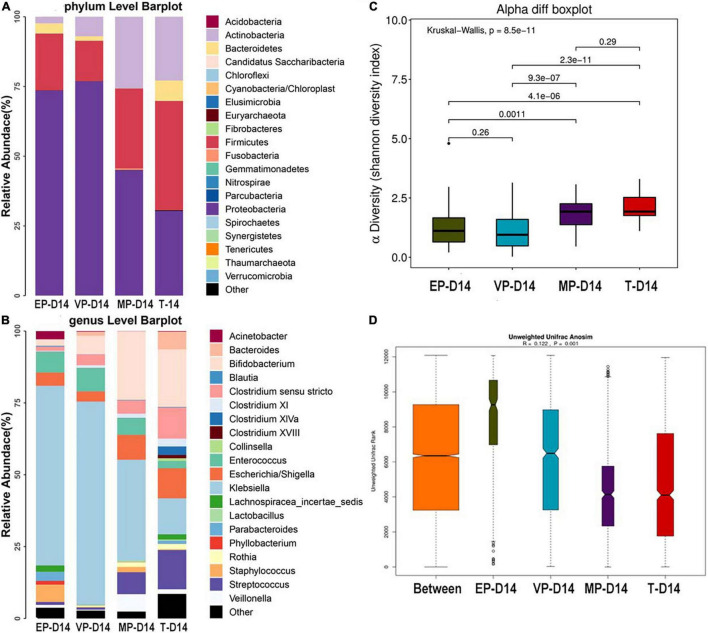
The composition of gut microbiota from neonates with different gestational ages at 14 days after birth. EP, extremely preterm; VP, very preterm; MP, moderate to late preterm; T, full-term. **(A)** Comparison of abundance at phylum level. **(B)** Comparison of abundance at genus level. **(C)** Shannon diversity index of α diversity. **(D)** Anosim analysis of β diversity.

The fecal microbiota composition of the preterm and term groups at the phylum and genus levels at day 14 after birth is shown in Figure 3. The gut microbiota composition at 14 days after birth was similar to that meconium in the EP group at the phylum level, because of the three most abundant phyla being Proteobacteria, Firmicutes, and Bacteroidetes. The third most abundant phylum in the VP and MP groups changed to Actinobacteria, while in the T group, the three most abundant bacteria at the phylum level were Firmicutes, Proteobacteria, and Actinobacteria (Figure 3A) and all above phyla were significantly different (Supplementary Table 2). At the genus level, the top three most abundant genera in the EP group were *Klebsiella* (62.5%), *Enterococcus* (7.2%), and *Staphylococcus* (5.9%), and those in the VP group were *Klebsiella* (70.8%), *Enterococcus* (8.1%), and *Bifidobacterium* (6.4%). Those in the MP group were *Klebsiella* (35.0%), *Bifidobacterium* (23.8%) and *Escherichia*/*Shigella* (8.6%), and those in the T group included *Bifidobacterium* (20.0%), *Streptococcus* (13.7%), and *Klebsiella* (12.5%). Also, except *Enterococcus* and *Escherichia/Shigella*, the left genera were significantly different (Supplementary Table 2). The abundance of *Bifidobacterium* in the VP, MP, and T groups gradually increased at 14 days after birth, while the abundance of *Bifidobacterium* in the EP group was still lower; however, the main genus in preterm groups was *Klebsiella* (Figure 3B). The Shannon index of alpha diversity (Figure 3C) showed no significant difference between the EP and VP groups, MP and T groups, the rest were significantly different. The ANOSIM index, reflecting beta diversity (showed *R* = 0.122 and *P* = 0.001) (Figure 3D), indicated that the four groups exhibited marked differences. The communities that mainly contributed to the T group were *Clostridiales* (order), *Clostridia* (class), and *Streptococcaceae* (family), while those in the MP group were *Bifidobacterium*, *Bifidobacteriales* (order), and *Bifidobacteriaceae* (family). Those in the VP group were *Klebsiella*, *Gammaproteobacteria* (class), and *Enterobacteriaceae* (family), and those in the EP group were *Bacillales* (order), *Staphylococcus*, and *Staphylococcaceae* (family) (Supplementary Figure 2).

#### Day 28 After Birth

**FIGURE 4:**
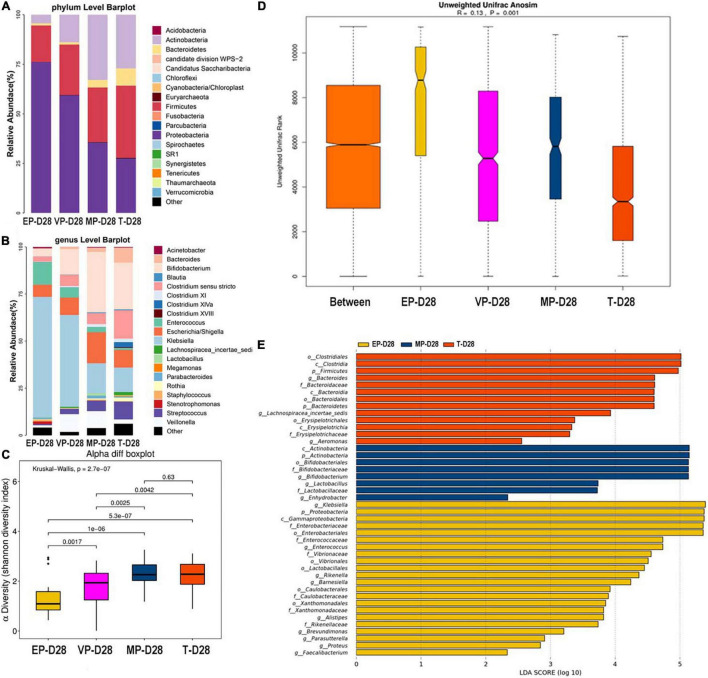
The composition of gut microbiota from neonates with different gestational ages at 28 days after birth. EP, extremely preterm; VP, very preterm; MP, moderate to late preterm; T, full-term. **(A)** Comparison of abundance at phylum level. **(B)** Comparison of abundance at genus level. **(C)** Shannon diversity index of α diversity. **(D)** Anosim analysis of β diversity. **(E)** Communities or species that have significantly different effects for each group. The abscissa represents LDA score. LDA score obtained by linear regression analysis of microbial groups with significant effects in different groups.

At 28 days after birth, the top three most abundant phyla in the EP and VP groups were Proteobacteria, Firmicutes, and Actinobacteria, which the gut microbiota composition was similar to that at day 14 in the VP and MP group at the phylum level. Those in the MP group were Proteobacteria, Actinobacteria, and Firmicutes. Those in the T group were Firmicutes, followed by Proteobacteria and Actinobacteria (Figure 4A). However, the abundance of above phyla among four groups were totally different (Supplementary Table 3). At the genus level, the top three most abundant genera in the EP group were *Klebsiella* (64.0%), *Enterococcus* (11.9%), and *Escherichia*/*Shigella* (6.5%). Those in the VP group were *Klebsiella* (48.7%), *Bifidobacterium* (13.6%), and *Veillonella* (9.4%), and those in the MP group were *Bifidobacterium* (32.1%), *Escherichia*/*Shigella* (16.5%), and *Klebsiella* (15.9%). Those in the T group were *Bifidobacterium* (24.8%), *Clostridium* (15.1%), and *Klebsiella* (13.0%). Except *Escherichia/Shigella* the left genera were significantly different (Supplementary Table 3). *Klebsiella* was still the main genus of the gut microbiota in the preterm groups. While the abundance of *Bifidobacterium* in the EP group was still low at 28 days after birth (Figure 4B). However, *Bifidobacterium* gradually enriched in VP, MP, and T group at 28 days after birth. There was no significant difference in alpha diversity between the MP and T group (Figure 4C). Beta diversity (Figure 4D), with *R* = 0.13 and *P* = 0.001, significantly differed among the groups. *Clostridiales* (order), *Clostridia* (class), and Firmicutes were the main contributors in the T group. Those in the MP group were *Actinobacteria* (class), Actinobacteria and *Bifidobacterium* (order), and those in the EP group were *Klebsiella*, Proteobacteria, and *Gammaproteobacteria* (class) (Figure 4E).

#### Day 120 After Birth

**FIGURE 5:**
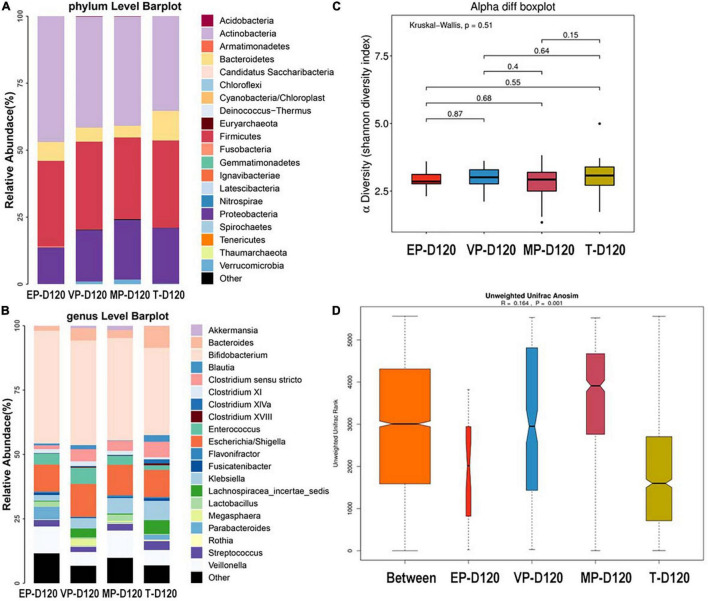
The composition of gut microbiota from neonates with different gestational ages at 120 days after birth. EP, extremely preterm; VP, very preterm; MP, moderate to late preterm; T, full-term. **(A)** Comparison of abundance at phylum level. **(B)** Comparison of abundance at genus level. **(C)** Shannon diversity index of α diversity. **(D)** Anosim analysis of β diversity.

The top three most abundant phyla of the four groups were Actinobacteria, Firmicutes, and Proteobacteria at 120 days after birth (Figure 5A). The abundance of Actinobacteria of four groups were significantly different (Supplementary Table 4). At the genus level, the top three most abundant genera of the EP and MP groups were *Bifidobacterium* (43.8, 39.8%), *Escherichia/Shigella* (10.54, 11.9%), and *Veillonella* (10.45, 10.6%). Those of the VP group were *Bifidobacterium* (40.6%), *Escherichia/Shigella* (12.8%), and *Enterococcus* (6.3%) and those for the T group were *Bifidobacterium* (33.9%), *Escherichia/Shigella* (10.6%), and *Bacteroides* (8.5%) (Figure 5B). The abundance of *Enterococcus* of four groups were significantly different, the abundance of *Bifidobacterium* of four groups were shown no markedly different at day 120 (Supplementary Table 4). The abundance of *Bifidobacterium* in the EP group increased from 28 days after birth to 120 days after birth, and increased in other groups too. There was no significant difference among the four groups in alpha diversity as shown in Figure 5C. Beta diversity was *R* = 0.164, *P* = 0.001 (Figure 5D), and the difference among groups was also significant. The communities that mainly contributed to the T group were *Clostridia* (class), *Clostridiales* (order), and *Lachnospiraceae* (family), and those for the MP group were *Lactobacillaceae* (family), *Lactobacillus*, and *Gemmiger* and those for the VP group were *Enterococcus, Enterococcaceae* (family), and *Megasphaera*. Those for the EP group were *Parabacteroides*, *Olsenella*, and *Clostridiales* (family) (Supplementary Figure 3).

#### Day 365 After Birth

**FIGURE 6:**
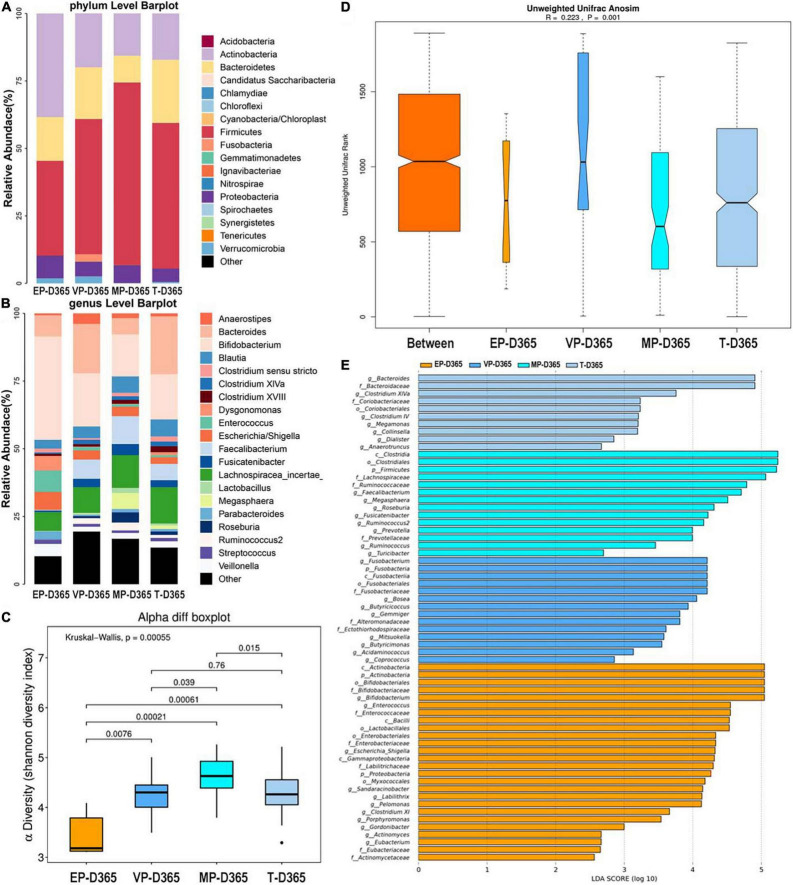
The composition of gut microbiota from neonates with different gestational ages at 365 days after birth. EP, extremely preterm; VP, very preterm; MP, moderate to late preterm; T, full-term. **(A)** Comparison of abundance at phylum level. **(B)** Comparison of abundance at genus level. **(C)** Shannon diversity index of α diversity. **(D)** Anosim analysis of β diversity. **(E)** Communities or species that have significantly different effects for infants with different gestational ages at 365 days after birth.

At 365 days after birth, the top three most abundant phyla of the EP group were Actinobacteria, Firmicutes, and Bacteroidetes. Those for the VP and MP groups were Firmicutes, Actinobacteria, and Bacteroidetes, and those for the T group were Firmicutes, Bacteroidetes, and Actinobacteria (Figure 6A). Firmicutes and Actinobacteria shown significantly different (Supplementary Table 5). At the genus level, the top three most abundant genera of the EP group were *Bifidobacterium* (38.1%), *Enterococcus* (7.9%), and *Bacteroides* (7.8%), and those for the VP group were *Bifidobacterium* (19.7%), *Bacteroides* (18.2%), and *Lachnospiracea* (9.5%). Those for the MP group were *Bifidobacterium* (15.5%), *Lachnospiracea* (12.1%), and *Faecalibacterium* (10.3%), and those for the T group were *Bacteroides* (21.3%), *Bifidobacterium* (16.7%), and *Lachnospiracea* (13.4%) (Figure 6B). Except *Lachnospiracea*, the left genera above were significantly different (Supplementary Table 5). The alpha diversity is shown in Figure 6C, there was no significant difference between the EP and T groups, while the other groups showed significant differences and the Shannon index of MP was significantly higher than other three groups. Beta diversity with *R* = 0.223 and *P* = 0.001 (Figure 6D), indicated significant differences among groups. The communities that mainly contributed to the T group were *Bacteroides*, *Bacteroidaceae* (family), and *Clostridium XIVa*, while those in the MP group were *Clostridia* (class), *Clostridiales* (order), and Firmicutes. Those in the VP group were *Fusobacterium*, Fusobacteria, and *Fusobacteriia* (class), while those in the EP group were *Actinobacteria* (class), Actinobacteria, and *Bifidobacteriales* (order) (Figure 6E).

### Principal Co-ordinates Analysis

**FIGURE 7:**
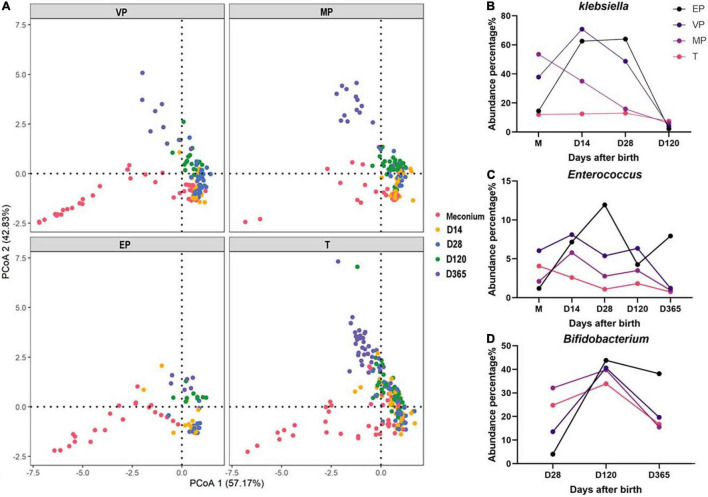
**(A)** PCoA of all samples in each group. **(B–D)** The dynamic changes of abundance for *Klebsiella*, *Enterococcus*, and *Bifidobacterium.*

All samples were combined, and PCoA was carried out. PCoA preliminarily reflects the distribution of different samples, which may show dispersion and aggregation. The results revealed that the dispersion of extremely preterm infants and term infants was similar in meconium, but at day 365 was completely different (Figure 7A). There was resemblance in the dispersion of groups VP and MP at days 14, 28, 120, and 365.

### Fecal Microbiome of Infants With Different Gestational Age at Different Days After Birth

The fecal microbiota structure of preterm infants in the same group at different days after birth was compared (Supplementary Figure 4). We found that at the phylum level, the abundance of *Proteobacteria* in the EP and VP groups first increased and then decreased, whereas it decreased continuously in the MP and T groups (Supplementary Figures 4A,C,E,G). The abundance of *Actinobacteria* increased from day 120 in the EP and VP groups. *Klebsiella* was highly abundant in the EP and VP groups until 28 days after birth (Figure 7B and Supplementary Figures 4B,D) and *Enterococcus* was enriched in the EP group at days 14–28 after birth (Figure 7C). Meanwhile, the abundance of *Bifidobacterium* increased until 120 days after birth in the EP group, while it remained stable in the MP and T groups from day 14 after birth (Figure 7D and Supplementary Figures 4F,H).

## Discussion

Complications in preterm infants are the leading cause of death in the first month of life and continue to affect the outcome of preterm infants (He et al., 2017; Cao et al., 2021; Bell et al., 2022). The gut microbiota plays important roles in health and disease. There is a critical period in early life when the gut microbiota composition is particularly malleable and influenced by various factors. Disruption to the microbiome during this period can be beneficial or detrimental to health (Ahearn-Ford et al., 2022). In order to summary the features, we profiled the gut microbiota of infants with different GAs at different postnatal time points.

As we know, the establishment of gut microbiome from neonates effect by variable factors, mode of delivery, feeding pattern, use of antibiotics, environment and so on (Healy et al., 2022). It is reported that in term infants c-section associates with stunted microbiota and colonization of opportunistic pathogen, including *Enterococcus*, *Enterobacter* and *Klebsiella* species (Shao et al., 2019). C-section also can delay the establishment of gut Microbiota in term infants (Kim et al., 2020). In our study, *Klebsiella* was significantly different in meconium among four groups. Meanwhile, delivery mode of four groups were significantly different (*P* < 0.001). It indicated that besides GA, gut microbiome of preterm infants can be greatly influenced by delivery mode.

With the growth of infants after birth, the situation of every infant became different and complex, especially for preterm infants. In our cohort of this research, the number of preterm infants with respiratory-related diseases such as NRDS, pneumonia and BPD obviously increased with the GA decreasing, which was significantly different among four groups. The number of infants with PVL and PDA was markedly different. The number of preterm infants with sepsis and NEC was not statistical difference, but morbidity of sepsis and NEC of preterm groups was higher than term group. Almost all preterm infants used different kinds of antibiotics in NICU. At the same time, especially extremely preterm and very preterm infants, most of their birth was due to the diseases of mothers, they always accepted mixed feeding pattern after birth that first were fed with formula milk and changed when their mother offered breast milk. It is well known that the gut microbiota is also strongly affected by feeding patterns and antibiotics (Healy et al., 2022). Bozzi Cionci et al. (2022) reported longtime exposure to antibiotics in the first weeks of in very preterm neonates affected *Clostridium* and *B. fragilis* levels and these changes did not persist at 90 days of life. In addition, NICUs harbor highly diverse bacterial communities and it is thought that bidirectional colonization between infants and NICU environment (Brooks et al., 2018). All above influenced the composition of gut microbiota of preterm infants. The gut microbiota of four groups were completely different from 2 weeks until 28 days after birth as a result.

The gut microbiota of preterm infants were gradually catching up with that of term infants 120 days after birth, because it showed that top three phylum and top two genus were the same between the preterm groups and term group which meant the bacteria composition was similarly, although the abundance was different. However, at 1 year after birth, the gut microbiota of preterm and term infants remained differed, not only the diversity of each group was different, but also the top three abundance of phylum and genus was different among four groups, and abundance were different too. Dynamic changes for gut microbiota of MP and T infants, the abundance of Proteobacteria decreased with the growth of neonates, but for EP and VP infants, the abundance of Proteobacteria first increased and then decreased. These differences between four groups especially from days 120 to 1 year after birth might be caused by feeding patterns, nature environment of family and infection situations of each infant after they left hospital.

At the genus level, the *Klebsiella* abundance in preterm infants was significantly different among four groups at different time points after birth. *Klebsiella* is a gram-negative bacterium, and one of the most common species is *Klebsiella pneumoniae*. Seki et al. (2021) reported that *Klebsiella* overgrowth in the gut is highly associated with brain damage in extremely preterm infants. *Klebsiella* is also associated with NEC (Paveglio et al., 2020), nosocomial infections, and LONS (Chen et al., 2020; Ma et al., 2021). van Best et al. (2020) observed that preterm infants (GA < 32 weeks) who received probiotics had increased abundance of *Bifidobacterium* and reduced abundance of *Klebsiella* and reduced monthly incidence of NEC. *Enterococcus* was significantly different from 28 days after birth till 1 year. *Enterococcus* was thought to be one of four phases and was only observed among EP infants and appeared to disturb the normal microbiota succession (Korpela et al., 2018). Furthermore, Al-Judaibi et al. (2021) reported that three common species were found in 15 of 19 stool samples of the preterm infants in the NICU, which were affected by commonly used antibiotics, with significant resistance of *Enterococcus* and *K. pneumoniae* to some of the tested antibiotics. *Enterococcus* is also a common pathogen in the NICU, and *Enterococcus* infection is the major cause of neonatal prematurity, meningitis, and sepsis (Reuschel et al., 2020). In another study, *Enterococcus* and *Streptococcus* were enriched in the non-probiotic formula-feeding group of premature infants (Chi et al., 2021). *Bifidobacterium* is a probiotic that confers health benefits on the host and its administration is regarded as a promising strategy to prevent neonatal NEC and sepsis (Hui et al., 2021). The abundance of *Bifidobacterium* showed the opposite trend of that of *Klebsiella* in our study. Except at 120 days, *Bifidobacterium* appeared significantly different among four groups at other time points. One study showed that probiotics, including *Bifidobacterium* supplementation in preterm infants, may prevent serious morbidities (Plummer et al., 2021). Another study showed that routine administration of a multispecies probiotic containing *Bifidobacterium* and *Lactobacillus* to very low birth weight infants had no significant impact on the NEC incidence (Juber et al., 2021) which shown the different results compared with reference Reuschel et al. (2020).

Comparing with term infants, the gut microbiota of preterm infants is always characterized by decreased microbial diversity (Chi et al., 2019; Henderickx et al., 2019; Jia et al., 2020). In our study, Shannon index for alpha diversity of meconium in EP group was significantly higher than other preterm groups and term group and Shannon index of meconium in preterm group was decreased with increasing of GA. Besides GA, some demographic characteristics of mothers in each group were significantly different, which suggested different alpha diversity of meconium were strongly associated with other situations not only GA. At day 14 to day 28 after birth, alpha diversity of T and MP group were significantly higher than other two preterm groups. However, alpha diversity of preterm groups and term group was no significant different at day 120 after birth, and MP group owned the highest alpha diversity at day 365 after birth which was higher than term group.

We tracked the development of the gut microbiota in preterm infants with different GA from newborn to 365 days after birth, compared it with that of term infants, and profiled the features of dynamic changes of gut microbiome. The results of our research provide new insights that we can interfere with specific microbes of preterm infants with different GA and different time points after birth, such as opportunistic pathogen *Klebsiella* and *Enterococcus*, as well as the probiotics *Bifidobacterium*.

Our study has some certain limitations. The number of fecal samples from EP and VP infants at day 365 was smaller than that in the other groups because of the weak compliance of the parents. Because of the limitation of sample size, we did not further divide by delivery mode, feeding pattern and antibiotic use. However, we carefully draw conclusions from our existing data, we will enlarge the sample size for validation in out further studies.

## Data Availability Statement

The data presented in the study are deposited in the NCBI repository with accession BioProject ID: PRJNA837453.

## Ethics Statement

The studies involving human participants were reviewed and approved by the Ethics Committee of Peking University Third Hospital. Written informed consent to participate in this study was provided by the participants or their legal guardian/next of kin.

## Author Contributions

XT: conceiving and design. XT and YX: supervising of study and critical revision of manuscript. YC, YY, and YW: subject recruitment and performance of clinical procedure. XY, ZC, BL, LC, and DM: bioinformatics analysis. QJ and XY: drafting of manuscript. All authors contributed to the article and approved the submitted version.

## Conflict of Interest

BL and LC were employed by National Engineering Center of Dairy for Maternal and Child Health, Beijing Sanyuan Foods Co., Ltd. The remaining authors declare that the research was conducted in the absence of any commercial or financial relationships that could be construed as a potential conflict of interest.

## Publisher’s Note

All claims expressed in this article are solely those of the authors and do not necessarily represent those of their affiliated organizations, or those of the publisher, the editors and the reviewers. Any product that may be evaluated in this article, or claim that may be made by its manufacturer, is not guaranteed or endorsed by the publisher.
