# Supplementary material for: Dynamic Changes of the Gut Microbiota in Preterm Infants With Different Gestational Age
Source: Front Microbiol. 2022 Jun 30;13:923273. doi: 10.3389/fmicb.2022.923273 (PMC9279133; doi:10.3389/fmicb.2022.923273)
Supplement: Supplementary file 1 [file Data_Sheet_1.zip › Supplementary Material/Supplementary Figures.docx]

# Supplementary figure legend


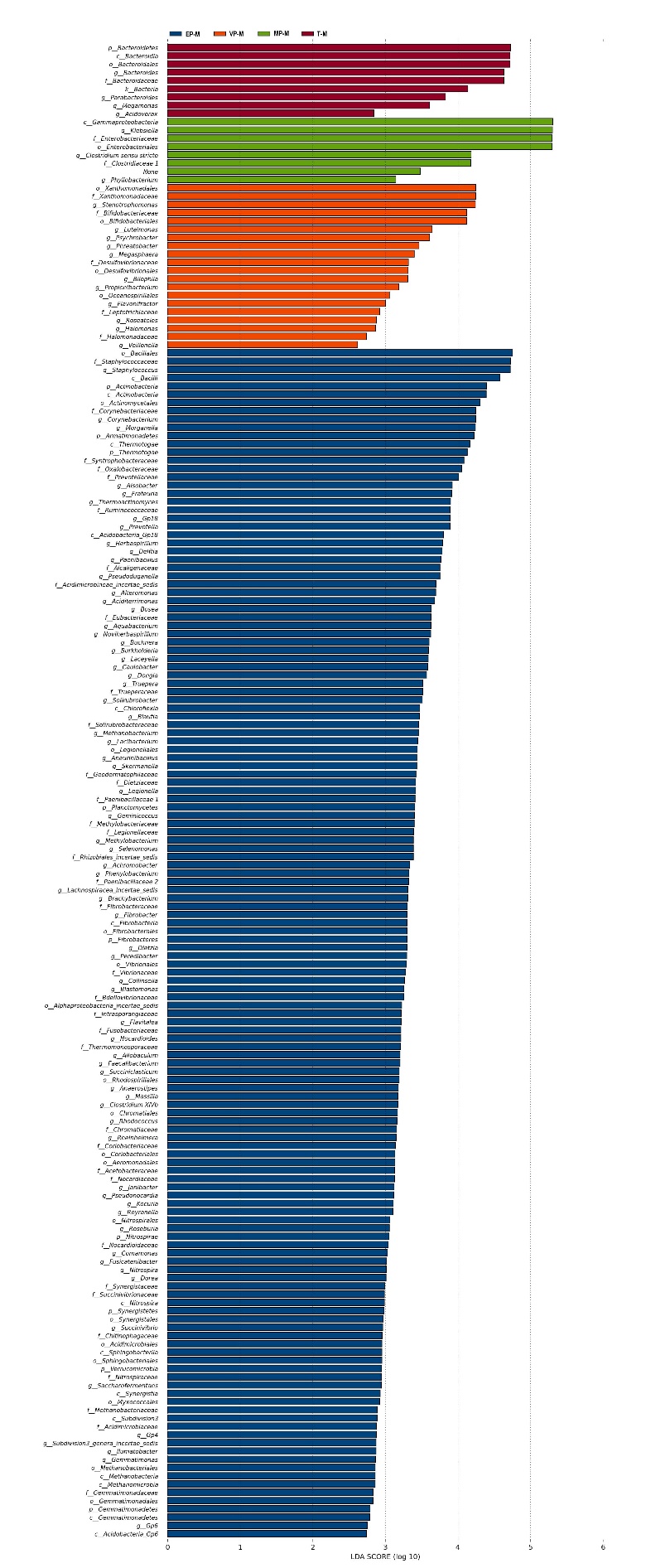


**Supplementary figure 1.** Communities or species that have significantly different effects for meconium of different gestational week infants. The abscissa represents LDA score. LDA score obtained by linear regression analysis of microbial groups with significant effects in different groups.


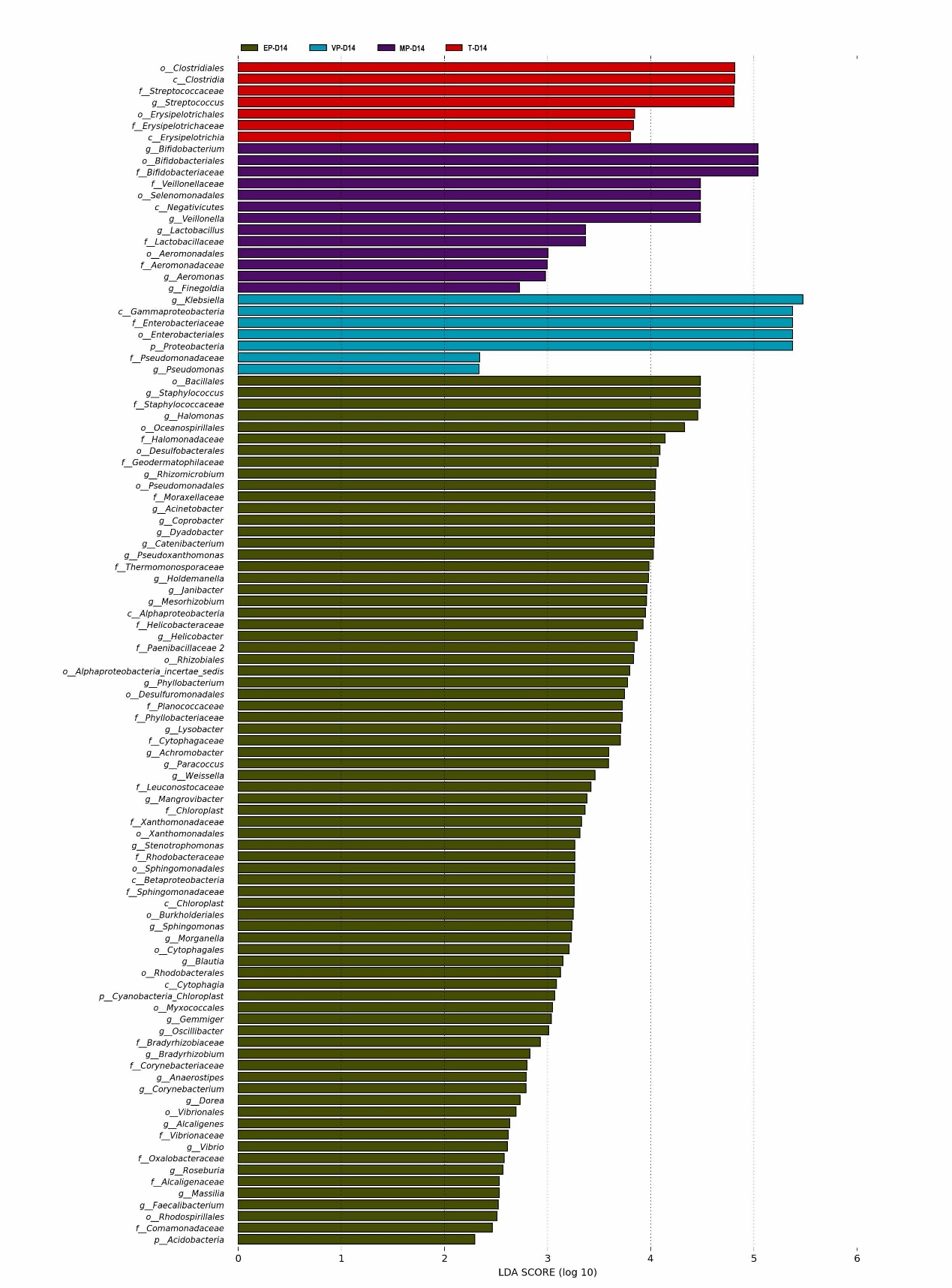


**Supplementary figure 2.** Communities or species that have significantly different effects for infants with different gestational ages at 14 days after birth.


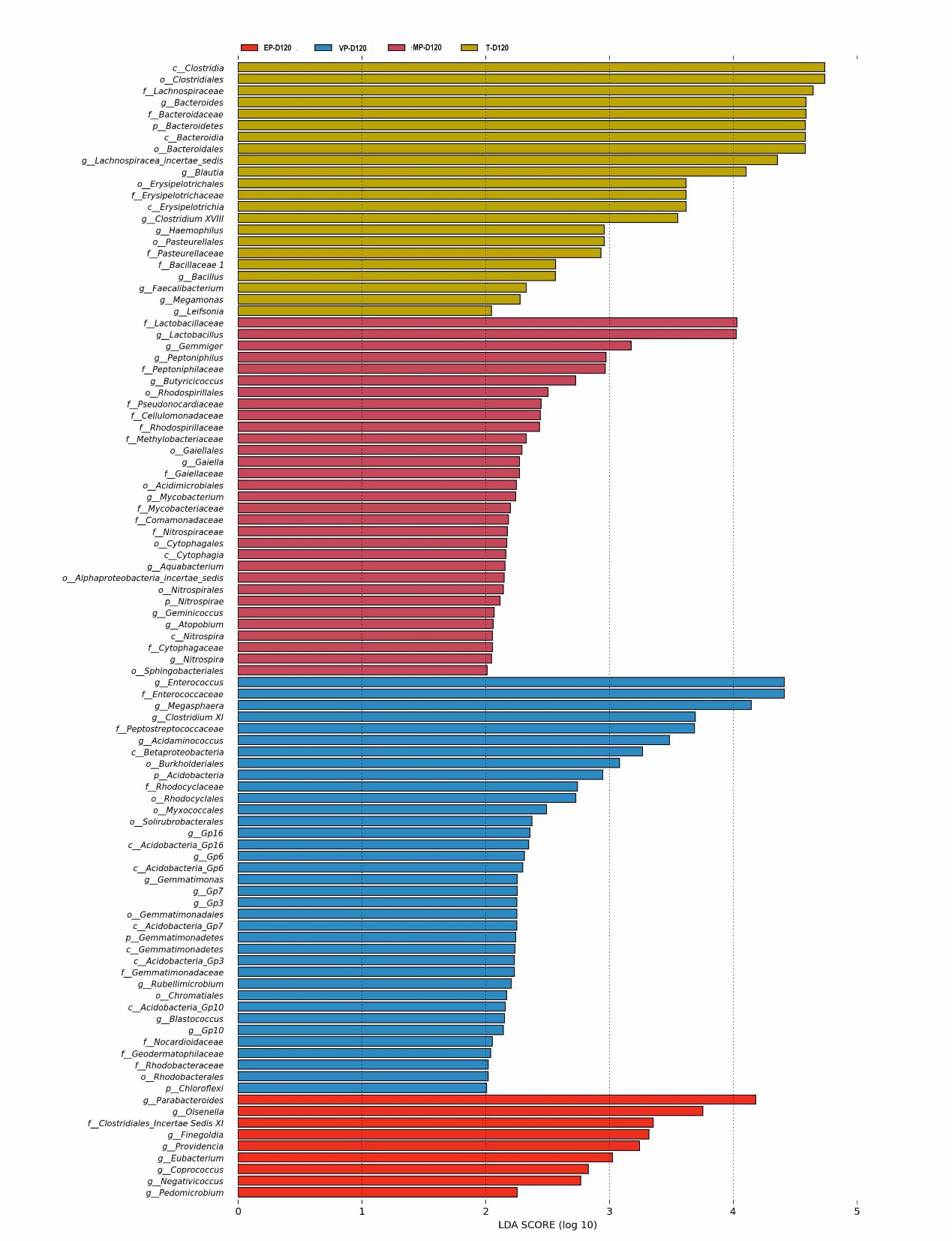


**Supplementary figure 3.** Communities or species that have significantly different effects for infants with different gestational ages at 120 days after birth.


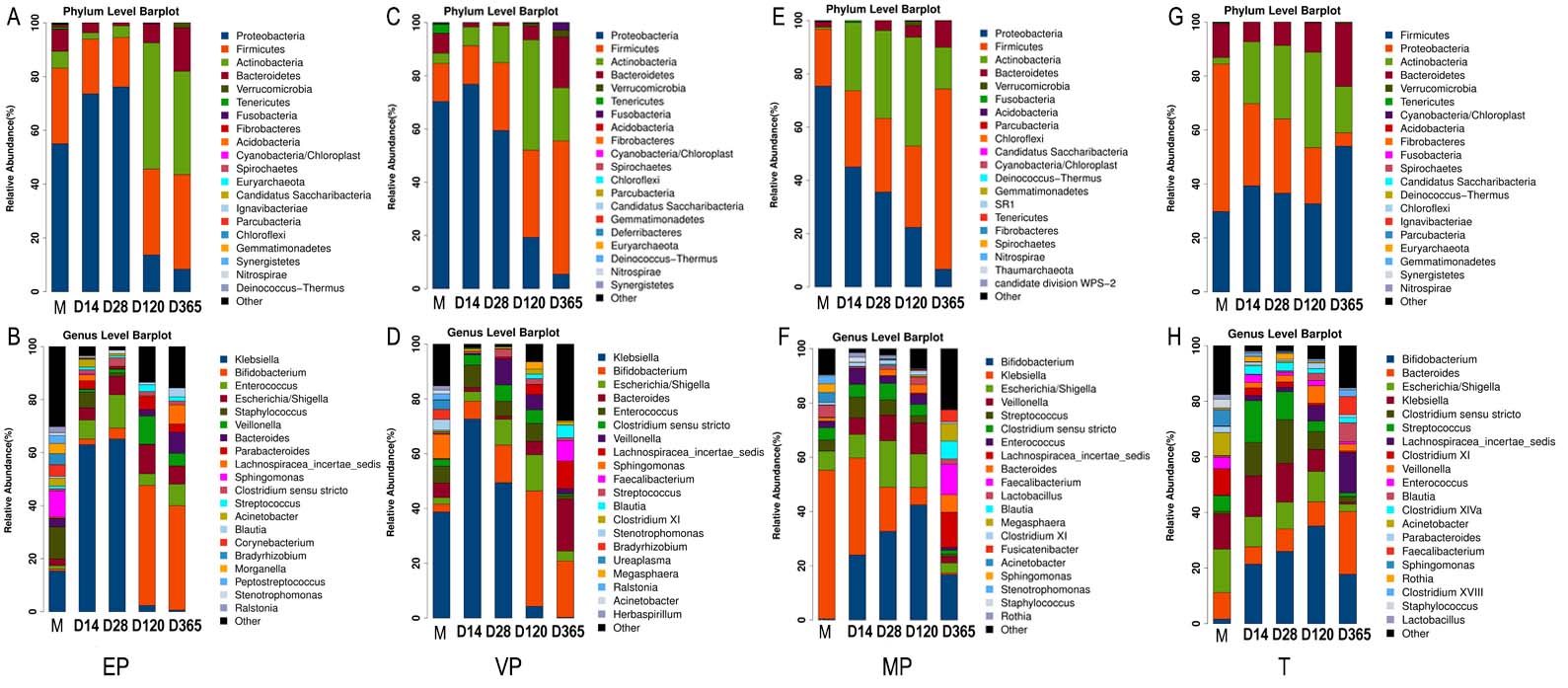


**Supplementary figure 4.** Comparison of abundance at phylum and genus level for different days after birth with different gestational ages. (A-B) phylum and genus level difference in extremely preterm group from birth to 365 days after birth. (C-D) phylum and genus level difference in very preterm group from birth to 365 days after birth. (E-F) phylum and genus level difference in moderate to late preterm group from birth to 365 days after birth. (G-H) phylum and genus level difference in full-term group from birth to 365 days after birth.
